# Engineering a feedback inhibition-insensitive plant dihydrodipicolinate synthase to increase lysine content in *Camelina sativa* seeds

**DOI:** 10.1007/s11248-021-00291-6

**Published:** 2021-11-20

**Authors:** Alex Huang, Cathy Coutu, Myrtle Harrington, Kevin Rozwadowski, Dwayne D. Hegedus

**Affiliations:** 1 Agriculture and Agri-Food Canada, 107 Science Place, Saskatoon, SK S7N 0X2 Canada; 2 Department of Food and Bioproduct Sciences, University of Saskatchewan, Saskatoon, SK Canada

**Keywords:** *Camelina sativa*, Lysine, Dihydrodipicolinate synthase, *Corynebacterium glutamicum*, Feedback inhibition

## Abstract

*Camelina sativa* (camelina) is emerging as an alternative oilseed crop due to its short growing cycle, low input requirements, adaptability to less favorable growing environments and a seed oil profile suitable for biofuel and industrial applications. Camelina meal and oil are also registered for use in animal and fish feeds; however, like meals derived from most cereals and oilseeds, it is deficient in certain essential amino acids, such as lysine. In higher plants, the reaction catalyzed by dihydrodipicolinate synthase (DHDPS) is the first committed step in the biosynthesis of lysine and is subject to regulation by lysine through feedback inhibition. Here, we report enhancement of lysine content in *C. sativa* seed via expression of a feedback inhibition-insensitive form of DHDPS from *Corynebacterium glutamicums* (CgDHDPS). Two genes encoding *C. sativa* DHDPS were identified and the endogenous enzyme is partially insensitive to lysine inhibition. Site-directed mutagenesis was used to examine the impact of alterations, alone and in combination, present in lysine-desensitized DHDPS isoforms from *Arabidopsis thaliana* DHDPS (W53R), *Nicotiana tabacum* (N80I) and *Zea mays* (E84K) on *C. sativa* DHDPS lysine sensitivity. When introduced alone, each of the alterations decreased sensitivity to lysine; however, enzyme specific activity was also affected. There was evidence of molecular or structural interplay between residues within the *C. sativa* DHDPS allosteric site as coupling of the W53R mutation with the N80V mutation decreased lysine sensitivity of the latter, but not to the level with the W53R mutation alone. Furthermore, the activity and lysine sensitivity of the triple mutant (W53R/N80V/E84T) was similar to the W53R mutation alone or the *C. glutamicum* DHDPS. The most active and most lysine-insensitive *C. sativa* DHDPS variant (W53R) was not inhibited by free lysine up to 1 mM, comparable to the *C. glutamicums* enzyme. Seed lysine content increased 13.6 -22.6% in CgDHDPS transgenic lines and 7.6–13.2% in the mCsDHDPS lines. The high lysine-accumulating lines from this work may be used to produce superior quality animal feed with improved essential amino acid profile.

**Supplementary Information:**

The online version contains supplementary material available at 10.1007/s11248-021-00291-6.

## Introduction

*Camelina sativa* (camelina)*,* an oilseed crop belonging to the *Brassicaceae* family, has emerged as a platform for aviation biofuel and various other industrial applications (Bansal and Durrett 2016). It is being developed as a sustainable rotation crop due to its short lifecycle (100–120 days) and reasonable productivity on marginal lands with low inputs (Vollmann and Eynck 2015). The seed oil contains 50–60% polyunsaturated fatty acids, of which 20–25% is omega 6 (linoleic acid, 18:2n-6) and 35–40% is omega 3 (linolenic acid, 18:3n-3) (Lu and Kang 2008). α-linolenic acid is the precursor for the essential long chain polyunsaturated fatty acids eicosapentanoic acid (20:5 ω3) and docosahexanoic acid (22:6 ω3) that have human health benefits. Camelina seed meal generated by cold-pressing is rich in protein (450 g Kg^−1^) and residual oil (100 g Kg^−1^) and can be used as a source of protein in farmed fish (Hixson and Parish 2014; Hixson et al. 2014; 2016a,b), poultry (Kakani et al. 2012), and livestock (Cappellozza et al. 2012; Colombini et al. 2014; Kahindi et al. 2014). Atlantic cod (*Gadus morhua*) tolerated up to 24% inclusion of camelina meal in place of fish meal in their diets without affecting weight gain (Hixson et al. 2016a); however, the levels of several essential amino acids are limiting as in most plant-based diets (Zubr 2003; Galili et al. 2016). Lysine and methionine, in particular, are often added as supplements to fish (Wilson and Halver 1986), poultry (Kidd et al. 1998) and swine feed (Brinegar et al. 1950) diets.

Improvement in the essential amino acid profile would increase camelina meal inclusion rates in feed. High-lysine corn varieties have been obtained through traditional breeding, as exemplified by the *opaque2* mutation (Mertz et al. 1964); however, this is linked to inferior agronomic traits. Improved Quality Protein Maize was successfully developed for commercial applications (Gibbon and Larkins 2005), but progress has been slow for other crops. Lysine content has been increased in cereal grains through transgenic expression of high lysine proteins, such as endogenous histones (Wong et al. 2015), BiP chaperone (Kawakatsu et al. 2010) or lysine-enriched fusion proteins (Yu et al. 2005; Chang et al. 2015; Liu et al. 2016; Jiang et al. 2016). Similar approaches have been taken with dicotyledonous species, including *Nicotiana tabacum* (Keeler et al. 1997), *Glycine max* (Zhang et al. 2014) and *Brassica napus* (Wang et al. 2011). Increased lysine content has also been achieved by suppressing the accumulation of low lysine seed storage proteins in *B. napus* (Kohno-Murase et al. 1995) and cereals (Kim et al. 2013; Schmidt et al. 2016).

Lysine and three other essential amino acids (methionine, isoleucine, and threonine) are derived from the aspartic acid pathway (Jander and Joshi 2010; Atkinson et al. 2012a; Galili et al. 2016; Wang et al. 2018). A crucial rate-limiting steps in lysine biosynthesis is catalyzed by the enzyme dihydrodipicolinate synthase (DHDPS; EC 4.2.2.52) (Galili 2002; Zhu and Galili 2003; Silk and Matthews 1997; Wang et al. 2018), which catalyses the condensation of pyruvate and (*S*)-aspartate semialdehyde (ASA) to form (4*S*)-4-hydroxy-2,3,4,5-tetrahydro-(2*S*)-dipicolinic acid (HTPA) (Blickling et al. 1997a). HTPA is further converted into lysine following reduction, amination, epimerization and decarboxylation reactions (Atkinson et al. 2013; Skovpen and Palmer 2013). Plant DHDPS enzymes are encoded by the *dapA* gene and localized to plastids (Ghislain et al. 1990) where their activity is regulated through feedback inhibition by free lysine (Jander and Joshi 2010) (Dereppe et al. 1992; Frisch et al. 1991; Ghislain et al. 1990; Kumpaisal et al. 1987; Negrutiu et al. 1984). Expression of lysine-insensitive DHDPS variants can increase accumulation of free lysine in plants (Frankard et al. 1992). Increased lysine levels in tobacco leaves (Ghislain et al. 1995) and the ability of a maize DHDPS to complement an *Escherichia coli dapA* mutation in the presence of an inhibitory lysine analouge (Shaver et al. 1996) were attributed to a lysine-insensitive DHDPS variants with single amino acid changes in their allosteric sites. The *E. coli* DHDPS is partially-insensitive to lysine and lysine content increased in tobacco seeds when it was co-expressed with a feedback-insensitive aspartate kinase (AK*, lysC*) (Karchi et al. 1994). The *Corynebacterium glutamicum* DHDPS (CgDHDPS, *CordapA*) is insensitive to feedback inhibition and total lysine content increased twofold in *B. napus* seeds when expressed alone and up to five-fold in soybean seeds when expressed in combination with AK (Falco et al. 1995). In rice, free lysine levels increased up to 12-fold in leaves and 60-fold in seeds in transgenic lines co-producing the *E. coli* AK and DHDPS in the chloroplast in combination with RNA interference to reduce levels of the catabolic enzyme lysine ketoglutaric acid reductase/saccharopine dehydrogenase (LKR/SDH) (Long et al. 2013). How this affected total lysine content was not determined. Similarly, production of a chloroplast-localized CgDHDPS and suppression of *LKR/SDH* expression increased free lysine by 40-fold in transgenic corn seed (Frizzi et al. 2008). In *Arabidopsis thaliana*, production of a bacterial lysine-insensitive DHDPS in a T-DNA insertion mutant of *LKR/SDH* synergistically increased free lysine levels 80-fold in the seed (Zhu and Galili 2003, 2004). Expression of CgDHDPS in a maize *opaque* mutant with reduced levels of the zein storage protein enhanced total lysine accumulation 1.3-fold in the grain (Huang et al. 2005). These data provide unambiguous evidence that manipulation of critical regulatory steps in the lysine biosynthetic pathway (AK, DHDPS and LKR/SDH) can lead to enhanced lysine accumulation in cereal grains and oilseeds. Here we examine the application of CgDHDPS and several engineered lysine-insensitive isoforms of an endogenous *C. sativa* DHDPS for increasing lysine levels in camelina meal.

## Materials and methods

### Plant materials and growth conditions

*Camelina sativa* (L.) Crantz line DH55, the source of the Genbank reference genome (Kagale et al. 2014; 2016), was used in this study. Plants used for phenotyping were grown in RediEarth (W.R. Grace & Co., Ajax, ON, Canada) in pots in a growth cabinet with the following settings: 16 h light/8 h dark and 20°C day/18 °C night temperature cycle.

### Seed germination and seedling growth assay

Seeds were surface-sterilized with 70% ethanol for 5 min and then with a 2.5% sodium hypochlorite solution (50% household bleach) for 7 min. After five washes in distilled water, seeds were placed on 0.5 X MS (Murashige and Skoog 1962; pH 6.0), 1% sucrose, 0.8% agar plates. Germination was recorded as the emergence of the radical. Seedling growth was monitored on the same media with or without 1.5 mM S-(2-aminoethyl)-L-cysteine (AEC; Sigma), a non-metabolizable analogue of lysine.

### Cloning and analysis the *DHDPS* gene from *C. glutamicum* and *C. sativa*

The *C. glutamicum DapA* (*CgDHDPS*) open reading frame (ORF) was amplified by polymerase chain reaction (PCR) from the genomic DNA of strain ATCC 130,302 (American Type Culture Collection) using KAPA HotStart DNA polymerase (VWR, Mississauga, ON, Canada) and two gene-specific primers: CgDHDPS-F (*Eco*RI) and CgDHDPS-R (*Bam*HI) (Supplemental Table S1). The resultant DNA was digested with *Eco*RI and *Bam*HI and ligated into pUC18 digested with the same restriction enzymes and then transformed into *E. coli* DH10B. *Camelina sativa* DH55 *DHDPS* cDNA was amplified from 100 ng leaf total RNA using the One-Step RT-PCR kit (Life Technologies) and two gene-specific primers: CsDHDPS F3 and CsDHDPS R3 (Supplemental Table S1). The resultant cDNA was purified and ligated into pGEM-T Easy (Promega) and transformed into *E. coli* DH10B. Several independent clones were sequenced to identify isoforms encoded by homoeologous genes. Of the three homoeologues in the camelina genome (Kagale et al. 2014; 2016), two were identified and used for further studies; these were denoted B4 (Csa16g004020) and B6 (Csa05g092770). Chloroplast targeting peptides were detected using the ChloroP 1.1 Server (http://www.cbs.dtu.dk/services/ChloroP-1.1/pages/output-expl.php).

### Site-directed mutagenesis of *CsDHDPS* cDNA

Mutations resulting in single amino acid changes were introduced into regions of CsDHDPS B6 within or adjacent to the allosteric site both independently and in combination. These were designated as CsDHDPS mA (W53R), CsDHDPS mB (N80V) and CsDHDPS mC (E84T) according to the numbering of residues in the *E. coli* DHDPS. *CsDHDPS B6* cDNA was cloned into pGEM-T Easy and site-directed mutagenesis (SDM) conducted using the Quick Change SDM kit (Life Technologies). Oligonucleotides used for cloning and mutagenesis are provided in Supplemental Table S1. Plasmid DNA was isolated from *E. coli* using the Qiaprep Spin Miniprep kit (Qiagen). Primers spanning the SDM site and *Pfu* Turbo high fidelity DNA polymerase (Agilent Technologies) were used to amplify the entire plasmid. *DpnI* is a restriction enzyme specific for methylated DNA and was used to remove the template plasmid leaving only the unmethylated plasmid DNA derived from the PCR. *Dpn*I-digested SDM reaction was used to transform *E. coli* DH10B and plasmids from positive colonies were sequenced to verify the mutations. To combine mutations, N80VB6F and N80VB6R SDM primers were used to integrate the N80V mutation (mB) into *CsDHDPS B6 W53R* (mA) to create the W53R/N80V double mutant (mAB). Similarly, the mBmC-F and mBmC-R SDM primers were used to integrate the N80V and E84T mutations into *CsDHDPS B6 W53R* to create the triple mutant (mABC).

### Complementation of the ***E. coli dapA***^***−***^ auxotroph with ***CgDHDPS ***and ***CsDHDPS***

Open reading frames encoding CsDHDPS B4 (364 aa; Csa16g004020) and CsDHDPS B6 (365 aa; Csa05g092770) or mutagenized variants were amplified from the pGEM-T Easy plasmids by PCR using *Pfu* Turbo DNA polymerase (Agilent Technologies) with CsDHDPS-F6 (*Eco*RI) and CsDHDPS-R6 (*Kpn*I) primers (Supplemental Table S1). The purified fragments were digested and inserted into the *Eco*RI and *Kpn*I sites of pUC18 in-frame with the *LacZ* ORF translation start codon. pUC18 plasmids harbouring the CsDHDPS variants or CgDHDPS were introduced into the *E. coli dapA*^*−*^ lysine auxotrophic strain AT997 (Genetic Stock Center, Yale University) which contains a defective *dhdps* gene and requires supplementation with lysine or diaminopimelic acid (DAP**)** to maintain normal growth on M9 minimum medium. Transformed colonies were isolated on M9 medium with 100 µM DAP and 100 µg/ml ampicillin at 37°C.

Growth was monitored on M9 medium alone, as well as M9 medium in the presence of 100 µM DAP or increasing concentrations of AEC (Sigma). Cells were grown in 10 ml of media in custom-built 50 ml Erlenmeyer flasks fitted with a 10 ml test tube sidearm at 37 °C with shaking at 225 rpm (Department of Chemistry, University of Saskatchewan). Cell culture was periodically transferred to the sidearm and optical density (A_610_nm) recorded with a digital colorimeter (Horizon 5965-50).

### DHDPS activity assay

DHDPS-containing cell extract was prepared using a procedure modified from Silk et al. (1994). Briefly, *E. coli* DH10B cells harboring the pUC18 DHDPS plasmids were grown in LB broth supplemented with 100 µM DAP, 14.8 µM thiamine hydrochloride, 1 mM MgSO_4_^.^7H_2_O, 11 mM D-glucose, 100 µg/ml ampicillin at 37°C with shaking (225 rpm). To induce *DHDPS* expression, 0.5 mM isopropyl β-D-1-thiogalactopyranoside (IPTG) was added to the cultures when the OD_600_ reached 0.6–0.8 and incubated for 6 h. Cells were harvested by centrifugation and pellets washed twice in buffer consisting of 50 mM Tris–HCl (pH 7.5), 1 mM EDTA, 1 mM 2-mecaptoethanol, 20% glycerol and 10 mM pyruvate, and re-suspended in 150 ul of fresh buffer. The cells were lysed by sonication for 30 s and stored at -20 °C. Total protein concentration was determined using a Q bit fluorimeter (Life Technologies).

DHDPS activity was measured as previously described (Vauterin et al. 2000) using L-aspartic-β-semi-aldehyde (L-ASA) as the substrate. Assays were carried out in 1 ml of buffer consisting of 100 mM Tris–HCl (pH 8), 35 mM pyruvate, 2 mM neutralized L-ASA, 40 µl cell extract and 35 µl of *o*-aminobenzaldehyde solution (0.5 mg *o*-ABA/35 µl ethanol). Reactions were incubated at 37^o^C for 30–90 min depending on the DHDPS activity in the reaction mixtures. The reactions were stopped by addition of 200 µl 12% trichloric acid (TCA) and placed in the dark. Under acidic conditions, *o*-ABA and L-2,3-dihydrodipicolinate react to form an adduct with a deep purple color (Yugari and Gilvarg 1965; Atkinson et al. 2012b), which develops maximally 2 h after TCA addition. The adduct is stable for about 6 h and its’ absorption is measured at 520 nm (Mitsakos et al. 2011; Erzeel et al. 2013; Atkinson et al. 2014). L-ASA was synthesized by Dr. D. Palmer in the Department of Chemistry, University of Saskatchewan, Canada. The effect of free lysine on DHDPS activity was assessed by inclusion of lysine in the reaction mixture at concentrations ranging from 12.5 µM to 10 mM depending on the sensitivity of the enzyme.

### Construction of binary vectors and plant transformation

DHDPS is localized to the chloroplasts of higher plants (Wallsgrove and Mazelis 1980), therefore, the amino-terminal chloroplast transit peptide (CTP) from the *A. thaliana* Rubisco small subunit (At1g67090) was appended to the CgDHDPS. The region encoding the CTP was amplified from *A. thaliana* genomic DNA using AtCTP-F and AtCTP-R and then re-amplified using CTP-F2 and CTPCgDS-R1 primers (Supplemental Table S1). The *CgDHDPS* open reading frame (ORF) was amplified from the pUC18 plasmid (above) using CTPCgDS-F1 and CgDS-R2 primers. The amplified fragments were purified and the CTP fragment was fused in-frame to the amino-terminus of CgDHDPS using SOEing PCR (Horton 1995) with gene-specific CTP-F2 and CgDS-R2 primers. All PCR reactions were performed with KAPA HiFi™ HotStart DNA polymerase (VWR). Following *Sal*I and *Not*I restriction digestion, the *CTP-CgDHDPS* fragment was amplified and cloned into the Gateway enter vector pENTR1A (Life Technologies). The *mCsDHDPS W53R* ORF was amplified by PCR and cloned into the *Kpn*I and *Not*I sites of pENTR1A. The *CTP-CgDHDPS* and *mCsDHDPS W53R* ORFs were transferred into the Gateway compatible binary vector pWY190 (Rozwadowski et al. 2008) via LR recombination. The vector uses the *Pap85* promoter to direct expression of the transgene and is active during seed maturation and early seed germination in *A. thaliana*. The *Streptomyces* species *PAT* (phosphinothricin acetyltransferase) gene under the control of the constitutive *tCUP2* promoter from tobacco (Wu et al. 2003) was used to provide selection for glufosinate (DL-phosphinothricin). The binary vector plasmids were transferred into *Agrobacterium tumefaciens* GV301 pMP90 and transformation of *C. sativa* DH55 (Kagale et al. 2014) was conducted using the floral dip method (Nguyen et al. 2014). Seed was provided by I. Parkin (Agriculture and Agri-Food Canada). Screening for positive glufosinate-resistant transformants was performed by spraying 6-day-old seedlings with 1.5 g L^−1^ Liberty herbicide (Bayer CropScience). Single-insert homozygous lines were selected using a droplet digital PCR (ddPCR) method (Comte et al. 2017) based on comparison of the copy number of the *PAT* gene with *CsActin2* as an internal reference.

Expression of the transgenes was verified using reverse-transcription PCR (RT-PCR). Total RNA was isolated from 5–10 mg of developing seed (30 days post-anthesis) using a protocol optimized for isolation of RNA from *B. napus* developing seeds (Sjödahl et al. 1993). RNase-free DNase I (Promega, Madison, WI, USA) was used to eliminate genomic DNA from total RNA prior to gene expression analysis using gene-specific primers (Supplemental Table S1) and the SuperScript one-step RT–PCR kit (Life Technologies) as described by Huang et al. (2009). The number of PCR cycles was optimized (30–35 cycles) so that the RT–PCR fell within the linear range. Transcripts from the *C. sativa 18S* ribosomal gene were amplified using Cs18S-F and Cs18S-R primers (Supplemental Table S1) and used as a normalization standard. Three biological replicates were conducted.

### Protein homology modeling

The theoretical structure of the *C. sativa* DHDPS isoform CsDHDPS-B6 was constructed based on target-template alignment using ProMod3 Version 1.1.0 within the Swiss-Model server (Biasini et al. 2014). Coordinates that were conserved between the target and the template were copied from the template to the model. Insertions and deletions were remodelled using a fragment library and side chains reconstructed. Finally, the geometry of the model was regularized by using GROMOS96 force field (Guex et al. 2009). The structure of DHDPS-2 from *A. thaliana* (PDB 4dpp) (Griffin et al. 2012) was identified as the best template by searching the SWISS-MODEL template library (SMTL version 2018-04-25, PDB release 2018-04-20) using BLAST (Camacho et al. 2009) and HHblits (Remmert et al. 2012). The 4dpp structure was determined by X-ray diffraction with a resolution of 2.00 angstroms. 4dpp shared a sequence identity of 90.52% and had a quaternary structure quality estimate (QSQE) of 0.71 with respect to the generated homology model CsDHDPS-B6. The CsDHDPS-B6 model conformed with the 4dpp template with a global mean quality estimate (GMQE) of 0.80 and a Qmean score of − 0.19 (Benkert et al. 2011). Imaging was done using UCSF Chimera (Pettersen et al. 2004).

### Amino acid quantification

Seeds were defatted with n-hexane based on the methods of Troeng (1955) and Barthet and Daun (2004). Mature seeds (2–3 g) were placed in sealed steel tubes with 3 ball bearings and 25 ml of hexanes (Sigma). Samples were ground for 45 min using an Eberbach shaker followed by vacuum filtration to remove oils and hexane. Defatted meal was air-dried overnight followed by storage at − 20°C. Total nitrogen content of the defatted meal was determined using a Flash EA 1112 Series N/Protein Analyser (Thermo Scientific). This system uses a dynamic flash combustion system coupled with a gas chromatographic separation system based on the AOAC Official Method 972.43 (1997). Approximately 15 mg of defatted meal from each sample was analyzed in triplicate. The nitrogen to protein conversion factor used was 6.25 (Jones 1931; AACC International Method 46-18.01 1999). Moisture levels in the defatted meal were determined as weight loss upon drying to stability (AACC Method 44-01.01 1999). Approximately 700 mg of defatted camelina meal was dried at 105°C for 24 h in a forced-air oven.

Amino acid profiles were analyzed following the procedure of AOAC Method 994.12 (2005) and Tuan and Philips (1997). Tryptophan was quantified following the method of Nielsen and Hurrell (1985). Microwave digestion methods were modified from Lill et al (2007) and Kabaha et al (2011). Separation and quantification of amino acids was performed using a high-performance liquid chromatography (HPLC) system (Waters Alliance 2695) equipped with a Waters 2475 fluorescence detector with excitation wavelength of 250 nm, emission wavelength of 395 nm and an AccQ-Tag C18 column for hydrolysate amino acid analysis, 3.9 × 150 mm (Waters). Gradient elution was diluted with AccQtag Eluent A buffer, water and acetonitrile. Amino acids were resolved using a multi-step gradient elution with an injection volume of 5 μl. Response peaks were recorded and analysed with Empower 3 software (Waters Corp.). Pre-column derivatization using AccQ-Fluor reagent (Waters Corp.) was done for all samples, except tryptophan which was diluted with an equivalent ratio of borate buffer and acetonitrile prior to HPLC analysis. For all amino acids except cysteine, methionine and tryptophan, 5 mg of sample (protein basis) was hydrolyzed with 6 M HCL (Optima grade, Fisher Scientific) with 1% (w/v) phenol in a 10 ml quartz hydrolysis tube using a microwave digester (CEM Discover SPD) set at a ramp time of 5.5 min, hold at 195°C for 10 min, maximum pressure of 140 psi and maximum power of 300 W. Hydrolysates were neutralized with sodium hydroxide, filtered through a 0.45 μm Phenex RC syringe filter to remove particulates and applied to a Waters Oasis HLB C18 Cartridge for sample cleanup prior to HPLC analysis. Amino acids were eluted from the cartridge with 5% (v/v) acetonitrile. Flow-through and washes were collected. Cysteine and methionine were determined as cystic acid and methionine sulfone after oxidation with performic acid followed by microwave hydrolysis with 6 M HCl, neutralization and filtration. Tryptophan was determined by hydrolyzing 10 mg of sample (protein basis) in 4.2 M NaOH in a 10 ml quartz hydrolysis tube with a Teflon liner using a microwave digester (CEM Discover SPD) set at a ramp time of 6.0 min, hold at 215°C for 20 min, maximum pressure of 140 psi and maximum power of 300 W. Hydrolyzed samples were neutralized with HCl and filtered prior to application on a Waters Oasis HLB C18 Cartridge. Tryptophan and 5-methyl tryptophan (internal standard) were eluted from the cartridge with 5% (v/v) acetonitrile/5% (v/v) methanol. The flow-through and washes were collected. Samples were stored at − 20°C prior to dilution and HPLC analysis. D,L 2-amino-butyric acid (0.1 mM) and D,L 5-methyl-tryptophan (0.1 mM) (Sigma-Aldrich) were used as internal standards and added to the sample hydrolysates following acid or base hydrolysis.

### Statistical analysis

For enzyme activity, Levene and Shapiro–Wilk tests were used to assess the homogeneity of variance and the normality at each treatment level. Differences between means of the lysine treatment levels within each line were identified using least squared regression followed by comparison with control (lysine = 0 μM) using the Dunnett’s test (JMP version 15.0.0; https://www.jmp.com/en_ca/home.html). Lines with probability of less than 0.05% were considered significant.

Percentages of each amino acid were calculated as a proportion of the total recovered amino acids, adjusted to a recovery of 100%. The Grubbs test was used to identify and remove outliers from the technical replicates prior to pooling for each biological replicate. Three biological replicates were used to evaluate the differences between means of all lines, for each amino acid, using standard least squared regression and ranked using the Tukey least significant difference test (LSD). P values less than 0.05% were considered significant.

Amino acid analysis and nitrogen analysis were performed in triplicate and moisture determination as a single sample. Technical replications presenting a large coefficient of variation (> 10) were repeated. Dunnett’s test or a one-way ANOVA with a multiple comparison Tukey HSD test were used to identify and rank significant differences.

## Results

### Isolation of *C. sativa DHDPS* cDNA

**Fig. 1:**
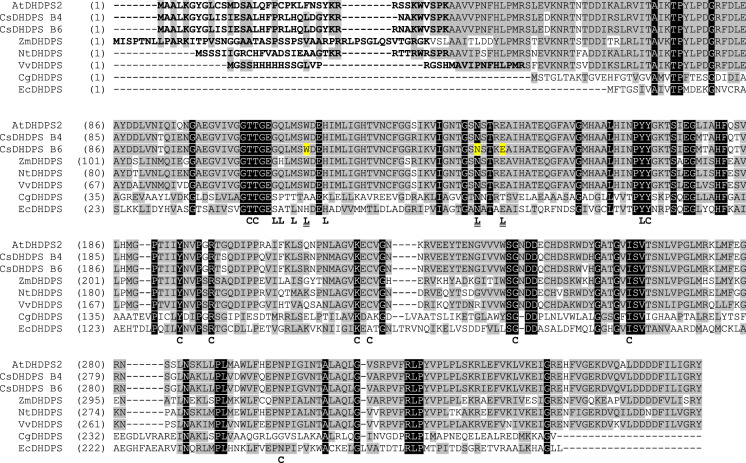
Conservation between *C. sativa* and other plant and microbial DHDPS enzymes. Amino acid sequence alignment of DHDPSs from *A. thaliana* (AtDHDPS2, Q9LZX6, 365 aa), *C. sativa* CsDHDPS B4 (Csa16g004020, 364 aa) and CsDHDPS B6 (Csa05g092770, 365 aa), *N. tabacum* (NtDHDPS, NP_001313049, 359 aa), *Z. mays* (ZmDHDPS, NP_001105425.1, 380 aa), *Vitis vinifera* (VvDHDPS, PDB 3TUU, 346 aa), *C. glutamicum* (CgDHDPS, X53993, 301 aa) and *E. coli* (EcDHDPS, WP_061350668, 292 aa). The predicated chloroplast transit peptides at the N-terminus in the plant enzymes are in bold text. Conserved amino acids involved in catalysis within the catalytic site (C) and those involved in lysine binding in the allosteric (L) sites are shown. The sites of the three mutations introduced into CsDHDPS B6 allosteric site (L and highlighted in yellow) are designated as mA (W53R), mB (N80V) and mC (E84T) according the numbering associated with the *E. coli* DHDPS. Amino acids found in all enzymes (white letters on black background) or amino acids with conserved properties (black letters on grey background) are shown. Alignment performed using the Vector NTI Suite (Life Technologies)

Two cDNAs encoding CsDHDPS, denoted B4 (Csa16g004020) and B6 (Csa05g092770), were identified and are homoeologues on sub-genomes I and III, respectively. Another *CsDHDPS* homoeologue (Csa07g004000) is present on sub-genome II, but did not appear among the cDNA clones sequenced. Csa05g092770 is closely linked to a truncated *DHDPS* gene (Csa05g092750) that may have arisen from a duplication event. Differences between the CsDHDPS B4 and B6 proteins included deletion of R^29^ within the chloroplast targeting peptide at the amino terminus of B4, and replacement of K^146^ and V^254^ in B6 with R^145^ and D^253^ in B4. A putative chloroplast targeting peptide was predicted to be located at the amino-terminus of all plant DHDPS enzymes (38 aa in *C. sativa*, 29 aa in *A. thaliana*, 32 aa in *N. tabacum*, 65 aa in *Z. mays*) (Fig. 1). Alignment of CsDHDPS B6 with other plant and bacterial DHDPS enzymes showed the plant DHDPSs shared variable identity with their *C. sativa* counterparts (94% with *A. thaliana*, 75% with *Nicotiana tabacum*, 67% with *Zea mays*), while the bacterial DHDPSs were more divergent (27% with *E. coli* and 22% with *C. glutamicum*).

Residues identified as crucial for catalytic activity of microbial DHDPS were conserved in the *C. sativa* DHDPS, including two residues near the amino-terminus (T^107^ and T^108^ corresponding to T^44^ and T^45^ in the *E.coli* DHDPS, respectively) and eight residues near the carboxy-terminus (Y^170^, Y^194^, R^199^, K^222^, C^224^, G^243^, I^261^, and N^300^ corresponding to Y^107^, Y^133^, R^138^, K^161^, A^163^, G^186^, I^203^ and N^248^ in the *E coli* DHDPS (Fig. 1). These residues have roles in the formation of the Schiff base (101-118, IPR020624; 194-224, IPR020625; http://www.ebi.ac.uk/interpro/entry), proton relay during catalysis (Y^107^ and Y^170^), pyruvate binding (T^108^ and I^261^) and reaction active sites (Y^194^, K^222^) (http://www.uniprot.org/uniprot/Q9LZX6). Notably, K^222^ (equivalent to K^184^ of the mature CsDHDPS protein) is conserved in the active site and plays a fundamental role in the ping-pong reaction (Atkinson et al. 2012a; Skovpen and Palmer 2013). As importantly, eight conserved residues involved in lysine binding are found in the allosteric regulatory domain (Fig. 1), whereupon binding of lysine leads to active site distortion or impaired dimer formation, resulting in inhibition enzyme activity (Laskowski et al. 2009). Structural modelling of the CsDHDPS indicates it forms a tetramer similar to the *A. thaliana* DHDPS (Griffin et al. 2012) (Supplemental Figure S1).

### Characterization of lysine-insensitive CsDHDPS variants

Previously, it was shown that a mutation in the *A. thaliana* DHDPS (W53R) resulted in the complete loss of feedback inhibition (Vauterin et al. 2000), while a N80I change in the *N. tabacum* DHDPS was responsible for lysine over-production (Ghislain et al. 1995). Similarly, the E84K substitution in the *Z. mays* DHDPS abolished its lysine sensitivity (Shaver et al. 1996). Both the *C. sativa* DHDPS B4 and B6 enzymes were able to complement the *E. coli dapA* mutation on minimal medium in the absence of DAP; however, the strain expressing DHDPS B6 exhibited more robust growth suggesting that it was more active. As such, site-directed mutagenesis was employed to generate similar mutations separately and in combination in the allosteric domain of *C. sativa* DHDPS B6. The three single amino acid changes were designated as CsDHDPS mA (W53R), CsDHDPS mB (N80V) and CsDHDPS mC (E84T) (Fig. 1; Supplemental Figure S1). Numbering of residues was according to *E. coli* DHDPS.

The auxotrophic *E. coli dapA*^−^ strain carrying only the pUC18 vector failed to grow on plates with M9 minimum medium in the absence of DAP; however, strains harbouring plasmids with the CgDHDPS, the wild-type (wt) CsDHDPS or any of the modified CsDHDPS variants grew to varying degrees indicating the modifications did not result in loss of DHDPS activity (Supplemental Figure S2). The addition of DAP restored growth of the *dap-* pUC18 strain and improved the growth of the lines carrying the wt CsDHDPS or variants. Lines carrying the CgDHDPS or the modified CsDHDPS variants also grew in the presence of the non-metabolizable lysine analogue AEC, indicating they were insensitive to feedback inhibition. Interestingly, AEC failed to completely inhibit the growth of the strain carrying the wt CsDHDPS after extended incubation (14 days) suggesting the endogenous plant enzyme may be partially insensitive to lysine inhibition. The CsDHDPS mA variant appeared to be least sensitive to AEC amongst the variants tested in this assay.

**Fig. 2:**
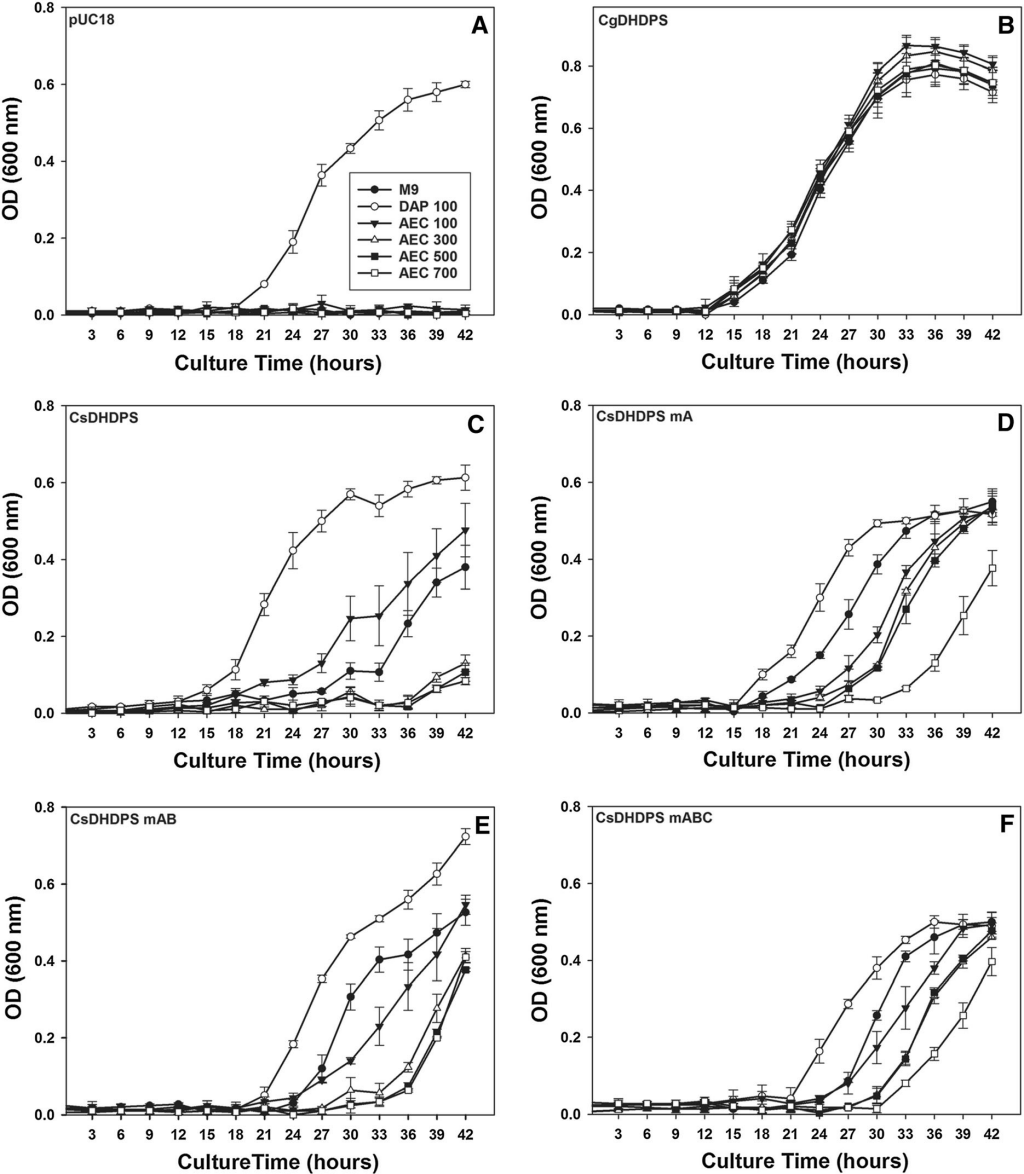
Growth of *E. coli* lysine auxotrophic (*dapA*^*−*^) strains harbouring plasmids expressing various DHDPS enzymes. pUC18 = empty vector control (Panel A), CgDHDPS = *C. glutamicum* DHDPS (Panel B), CsDHDPS = wt *C. sativa* DHDPS B6 (Panel C), CsDHDPS mA = W53R mutant (Panel D), CsDHDPS mAB = W53R/N80V double mutant (Panel E), CsDHDPS mABC = W53R/N80V/E84T triple mutant (Panel F). Growth medium included M9 minimal medium (M9), M9 + 100 μM diaminopimelate (DAP 100) or M9 with various concentrations (μM) of S-(2-aminoethyl)-L-cysteine (AEC). Errors bars show standard deviation (n = 3)

An assessment of the impact of the changes on DHDPS function was conducted by monitoring the growth of *E. coli dap-* strains expressing the DHDPS variants in the presence of AEC. The *E. coli dap-* pUC18 strain failed to grow in the absence of DAP (Fig. 2). The strain expressing the wt CsDHDPS grew on minimal M9 medium, although faster in the presence of 100 µM DAP, indicating the CsDHDPS enzyme is functionally equivalent to that encoded by the *E. coli dapA* locus. Growth of this strain was progressively impaired by increasing AEC concentrations, but was detectable even at the highest concentration suggesting that CsDHDPS may be partially insensitive to lysine. Strains expressing the CsDHDPS mA, mB or mC variants exhibited better growth than that expressing the wt CsDHDPS with only a short delay in the onset of the exponential phase and growth rates similar to that on M9 medium alone thereafter. The growth of the strain expressing CgDHDPS was not affected by any of the AEC concentrations tested. The time required for cultures to reach the same optical density (OD_600_ = 0.3) was also calculated to assess the in vivo sensitivity of modified DHDPS variants to AEC (Supplemental Table S2.1). In the absence of AEC and with the inclusion of DAP, the variants grew normally and reached similar culture densities (OD_600_ = 0.3) within 22–28 h. AEC addition increased the time for the variant cultures to reach the same density; however, strains expressing the CgDHDPS or CsDHDPS mA took the least time, 21.5–22 h and 31.5–40 h, respectively, indicating that these enzymes are the least sensitive to AEC or lysine. Growth rate during the exponential phase decreased in the strain carrying the wt CsDHDPS with increasing AEC concentration; however, growth rates were similar in the lines carrying the CsDHDPS variants once the exponential phase had begun (Supplemental Table S2.2).

**Fig. 3:**
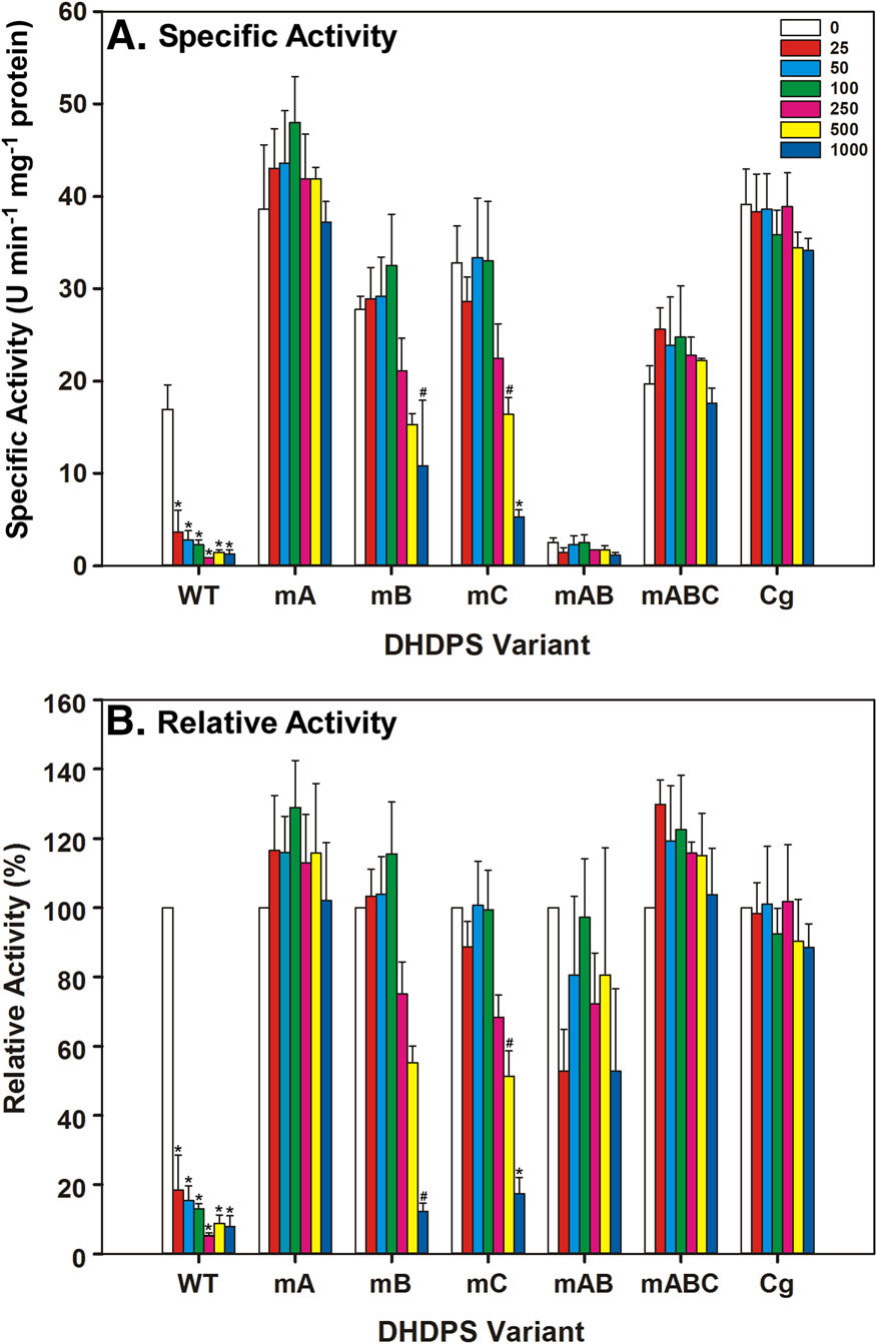
Sensitivity of various DHDPS enzymes to lysine feedback inhibition. Panel A: Specific activity of protein extracts from *E. coli* lysine auxotrophic (*dapA-)* strains harbouring plasmids expressing various DHDPS enzymes. C*. sativa* DHDPS B6 (Cs), CsDHDPS W53R mutant (mA), CsDHDPS N80V mutant (mB), CsDHDPS E84T mutant (mC), CsDHDPS W53R/N80V double mutant (mAB), CsDHDPS W53R/N80V/E84T triple mutant (mABC), *C. glutamicum* DHDPS (Cg). Panel B: DHDPS activity relative to activity in the absence of lysine. Lysine concentrations are in µM. Values that were significantly different from the control (0 lysine) within each group are indicated by an asterisk (*, *P* ≤ 0.05) or pound sign (#, *P* ≤ 0.10) according to Dunnett’s test. Error bars indicate standard deviation (n = 3)

In vitro enzyme assays were conducted to compare the degree to which the CsDHDPS variants were insensitive to lysine (Fig. 3). The specific activity of the single mutation mA, mB and mC variants was higher than that of the wt CsDHDPS and mA was comparable to that of CgDHDPS. The mAB combination significantly reduced activity, while the mABC combination displayed activity comparable to the wt CsDHDPS enzyme. The wt CsDHDPS was highly sensitive to lysine with only 20% of the activity remaining at 25 µM free lysine; the lowest concentration tested. A basal level of activity (ca. 10%) remained even at higher lysine concentrations which supports the tenet that the CsDHDPS enzyme may be partially insensitive to lysine. The mA CsDHDPS variant was the most insensitive to lysine inhibition with activity being unaffected even at the highest concentrations tested, 1000 µM free lysine, suggesting a critical role of the W53R substitution in lowering the affinity of the allosteric site for lysine. The mB and mC variants were more sensitive to lysine than the mA variant with activity diminishing as free lysine concentration increased and with less than 20% remaining at the 1000 µM lysine level. The double mutant (mAB) and the triple mutant (mABC) were less sensitive to lysine with the latter being comparable to the mA variant. The CgDHDPS was insensitive to lysine at all concentrations tested.

### Evaluation of transgenic *C. sativa* lines expressing *CgDHDPS *or *CsDHDPS mA*

**Fig. 4:**
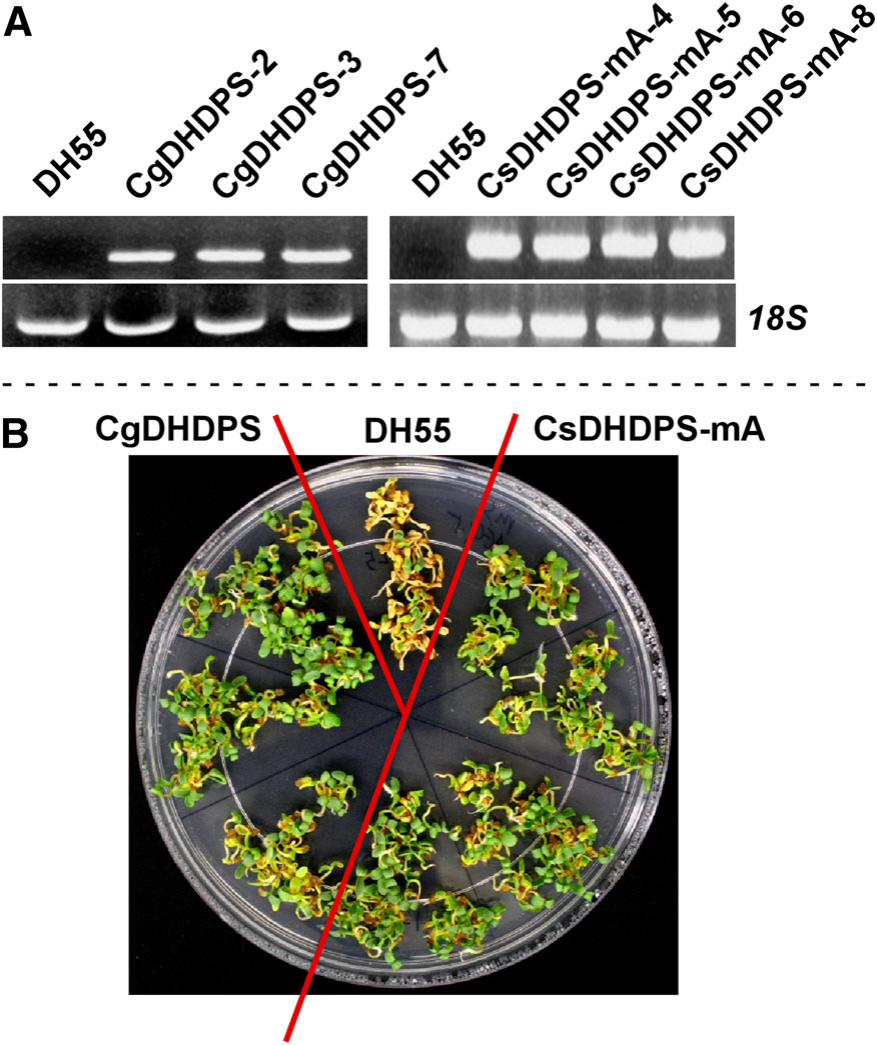
Characterization of transgenic *C. sativa* lines expressing genes encoding *C. glutamicum* DHDPS or the *C. sativa* DHDPS W53R mutant mA. Panel A: Gene expression as determined by RT-PCR in *C. sativa* DH55 (untransformed control) and several independent, single transgene insert, homozygous lines expressing *CgDHDPS* or *CsDHDPS mA*. Expression of *C. sativa* 18S ribosomal RNA was used as a control for total RNA input. Panel B: Growth of seedlings on 0.5 × MS-sucrose plates with 1.5 mM AEC

The most active and lysine-insensitive DHDPS enzymes (CgDHDPS and CsDHDPS mA) based on the in vitro and in vivo assays were selected for expression in *C. sativa* seeds. Three independent homozygous single-transgene lines expressing *CgDHDPS* and four expressing *CsDHDPS mA* (Fig. 4) were used to evaluate phenotypic changes and amino acid content.

The *PAP85* promoter is active during seed maturation and the early phase of seedling establishment (Rozwadowski et al. 2008). We confirmed that the CgDHDPS and CsDHDPS mA enzymes were active during this time by germinating seeds and growing the seedlings in the presence of AEC (Fig. 4). In the wt DH55 line, radicles initially emerged from the seed, but further growth was suspended and seedlings became chlorotic after 7 days and necrotic (brown) after 10 days. In contrast, transgenic seedlings expressing *CgDHDPS* or *CsDHDPS mA* were much lesser sensitive to AEC.

**Fig. 5:**
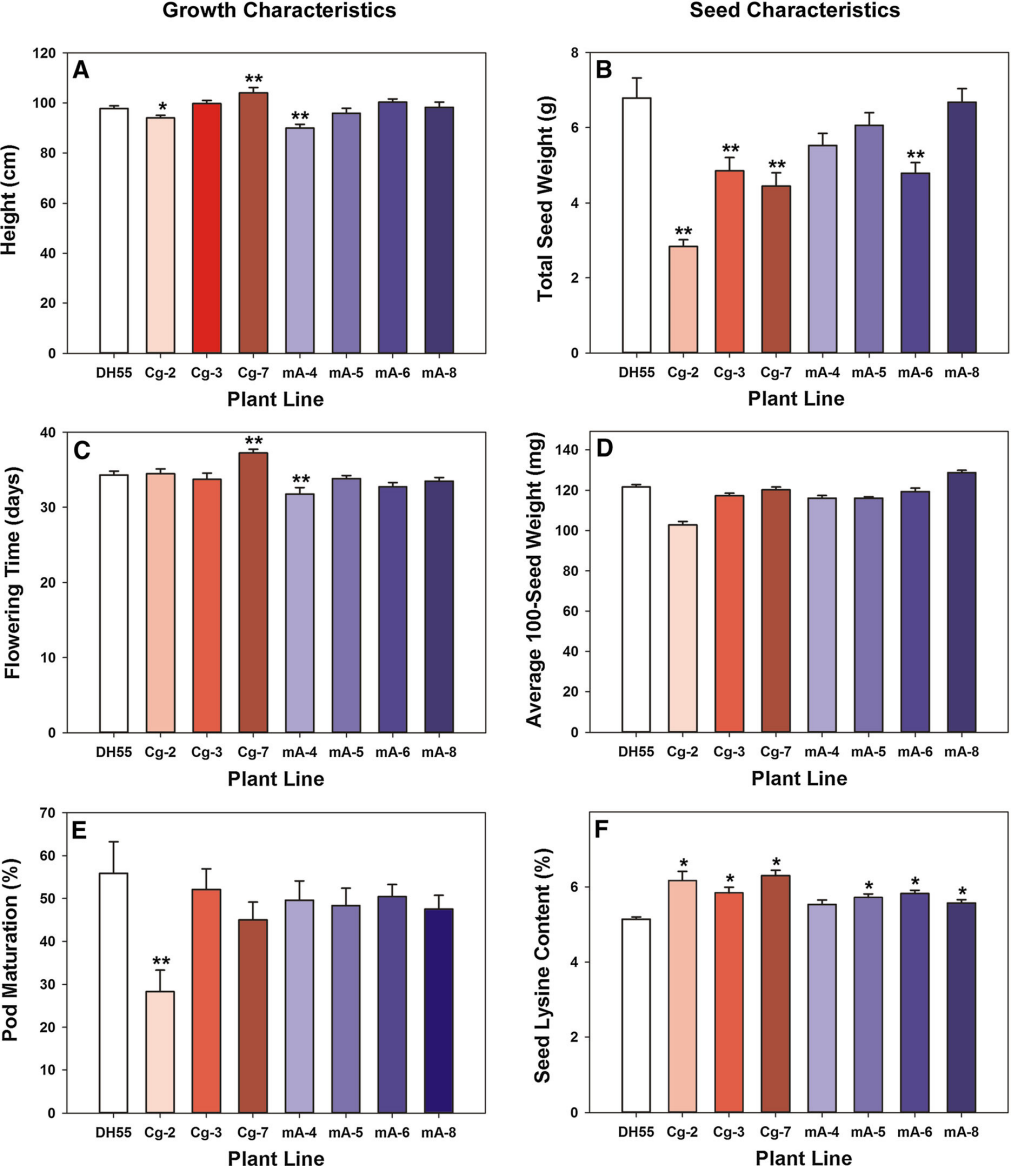
Comparison of growth (Panels A, C, E) and seed characteristics (Panels B, D, F) of *C. sativa* DH55 (untransformed control) to several independent, single transgene insert, homozygous lines expressing genes encoding *C. glutamicum* DHDPS (Cg) or the *C. sativa* W53R DHDPS mutant mA. Plant height was measured at 62 days, flowering time was recorded when first flower opened, pod maturation was determined at 77 days. Values that were significantly different from the control (DH55) are indicated by a single (*P* ≤ 0.05) or double (*P* ≤ 0.01) asterisk (one-way ANOVA and Tukey HST test). Error bars indicate standard deviation (n = 3)

Some alterations in plant growth and seed characteristics were noted when the CgDHDPS and CsDHDPS mA lines were grown in soil under controlled conditions. Seedlings of some of the transgenic lines were smaller and slower to establish relative to DH55 (Supplemental Figure S3), but only minor variations in plant height and flowering time were observed among the independent transgenic lines (Fig. 5). The CgDHDPS Cg-7 line was delayed in flowering (37.25 d), whereas the CsDHDPS mA-4 line flowered earlier (31.75 d) compared to DH55 (34.3 d). Pod maturation was not significantly delayed in the transgenic lines, except for the CgDHDPS Cg-2 line (Fig. 5). Total seed weight (yield) was reduced in all three CgDHDPS lines with total production only 42–71% of the DH55 control. Seed production was less affected in the CsDHDSP mA lines, which showed yields ranging from 70–98% that of DH55 (Fig. 5) and only one (mA-6) had significantly reduced total seed yield. None of the lines exhibited significantly different hundred seed weight compared to the DH55 control.

### Alteration of lysine and other amino acids in seed

Meal amino acid profile is governed mainly by the seed protein fraction, while free amino acids account for only 1–10% of the total amino acid pool. Therefore, the total (free and protein-associated) amino acid profiles were determined in the transgenic seed. All of the lines expressing *CgDHDPS* had significantly higher levels of lysine (5.84–6.3%) than the DH55 control (5.14 ± 0.06%; 26.74 ± 0.22 mg per g dry defatted meal), while three of the four lines expressing *CsDHDPS mA* lines had significantly greater levels of lysine (5.57–5.82%) (Supplemental Table S3). This represented a 13.6 to 22.6% increase in lysine with CgDHDPS and 7.6 to 13.2% increase with CsDHDPS mA compared to the DH55 control. Interestingly, lines that accumulated lysine at 13% or more above the DH55 level (CgDHDPS-2, -3, -7; CsDHDPS mA-6) also had reduced total seed yield (Fig. 5).

Most monogastric animals are unable to synthesize histidine, isoleucine, leucine, lysine, methionine, phenylalanine, threonine, tryptophan and valine, and must acquire them from their diets, hence these are referred to as essential amino acids. As such, the impact of increasing lysine content on the levels of these and other amino acids was also evaluated in the meal. In addition to lysine, histidine levels also increased in the transgenic seed by 3.7–6%, while the level of the non-essential amino acid alanine increased in CgDHDPS-2 (4%) and CgDHDPS-7 (3%), and the CsDHDPS mA-4 (2.8%) lines (Supplemental Table S3). In contrast, significant decreases were noted in tryptophan in CgDHDPS-3 (13.6%), CsDHDPS-mA-5 (12%) and CsDHDPS-mA-6 (12%). Reductions in tryptophan in other lines exceeded 7%, but were not considered statistically different from DH55. The sulphur-containing cysteic acid was significantly reduced in the CgDHDPS-3 (10.6%), CgDHDPS-7 (10%), CsDHDPS mA-4 (13.6%), CsDHDPS-mA-5 (10.9%) and CsDHDPS mA-4 (10.9%) lines. Arginine was also significantly reduced, but to a more moderate extent in CgDHDPS-3 (8%), CsDHDPS-mA-4 (3%), CsDHDPS-mA-5 (5%), CsDHDPS-mA-6 (6%) and CsDHDPS-mA-8 (6%). Lastly, a reduction in glutamic acid content was observed in CgDHDPS-2 (3.4%), but not in other transgenic lines.

## Discussion

Cold pressing of camelina seed results in a meal that is rich in protein and residual oil. Similar to other Brassicaceae, the meal contains anti-nutritional/anti-palative compounds such as glucosinolates, phytic acid and sinapine; however, it has been subjected to comparatively limited breeding efforts to address reducing these seed constituents. Even in its current form, camelina meal can be included in poultry (Kakani et al. 2012), swine (Kahindi et al. 2014), beef (Cappellozza et al. 2012; Colombini et al. 2014) and aquaculture (Hixon and Parish 2014; Hixon et al*.* 2014, Hixon et al. 2016a, 2016b) diets. Most plant-based diets are limiting in one or more essential amino acids, such as methionine and lysine (Zubr 2003; Galili et al. 2016), although camelina meal has a somewhat better constitution. In this study, a bacterial and an engineered isoform of *C. sativa* DHDPS were used to enhance lysine levels incorporated into protein in camelina meal.

Plant DHDPS enzymes are generally highly sensitive to lysine feedback-inhibition; however, isoforms from *A. thaliana* (Vauterin et al. 2000), *N. tabacum* (Ghislain et al. 1995) and *Z. mays* (Shaver et al. 1996) have been identified in which single point mutations greatly alter sensitivity to lysine. When engineered into *C. sativa* DHDPS, each of these mutations rendered the enzymes less sensitive to lysine feedback inhibition to varying degrees. W^53^ is present in the allosteric site and is conserved among plants, but not bacterial, DHDPS enzymes. It functions in the positioning of helix α2 similar to H^53^ in *E. coli* DHDPS (Blickling et al. 1997b). In the current study, introduction of a W^53^R mutation in *C. sativa* DHDPS resulted in a near complete loss of feedback inhibition while coincidently increasing its specific activity. In fact, this mutation resulted in a level of lysine insensitivity similar to that of the *C. glutamicum* DHDPS.

Feedback inhibition of DHDPS is mediated by the interaction of lysine with moieties within an allosteric site, which subsequently alters the conformation of the active site. The *C. glutamicum* DHDPS is impervious to lysine-feedback inhibition (Rice et al. 2008), while the *E. coli* DHDPS is partially insensitive to lysine, although the precise differences responsible for this are not fully understood (Geng et al. 2013). Comparison of lysine-bound and lysine-free grape vine DHDPS structures revealed a conformational shift within the allosteric site (Trp^78^) and important catalytic residues within the active site (Tyr^131^, Tyr^132^) upon lysine binding (Atkinson et al. 2013). Molecular dynamic simulations suggested the rotation of Tyr^132^ could attenuate proton relay through the catalytic triad in the presence of lysine. There was some indication of molecular or structural interplay between residues within the *C. sativa* DHDPS allosteric site as coupling of the W^53^R mutation with the N^80^V mutation decreased lysine sensitivity compared to N^80^V alone, but not to the level observed with the W^53^R mutation alone. However, when the E^84^T mutation was introduced into this double mutant to create a triple mutant, lysine sensitivity was similar to the W^53^R mutation alone or *C. glutamicum* DHDPS.

Each of the mutations increased lysine insensitivity, but also affected the activity of the enzyme when introduced alone. The same observation was made with *C. glutamicum* DHDPS isoforms in which the allosteric site had been altered to resemble that of *E. coli* (Geng et al. 2013) indicating even in the absence of lysine, residues within the allosteric site and their interaction impact the conformation of the active site. In support of this notion was the observation the specific activity of the CsDHDPS double mutant (W^53^R/N^80^V) was reduced relative to the wild type enzyme; however, activity was restored in the triple mutant (W^53^R/N^80^V/E^84^T). This is in agreement with structural studies indicating N^80^ is connected to R^138^ in the active site, therefore, any perturbation could have an immediate and profound impact on activity (Blickling et al. 1997a, 1997b).

In this study, the introduction of feedback-insensitive isoforms of DHDPS resulted in an increase in total seed lysine. While strategies to deregulate lysine biosynthesis or prevent its degradation lead to large increases in free lysine in seeds, this does not necessarily translate to equivalent increases in total lysine (free and incorporated into protein). For example, expression of *CordapA* in *B. napus* seeds led to a 100-fold increase in free lysine, but only a two-fold (100%) increase in total lysine (Falco et al. 1995) which is more than, but at least comparable to, that observed in the current study with *C. sativa* (22.6%). Furthermore, the amount of any free amino acid is very low compared to that incorporated into protein; therefore, reporting only increases in the free form can be misleading. Incorporation of free lysine into protein is dependent on the availability of uncharged lysyl-transfer RNAs and mRNA with corresponding codons. As such, further increases in total lysine accumulation could be achieved through the introduction of high-lysine sink proteins (Yu et al. 2005; Chang et al. 2015; Liu et al. 2016; Jiang et al. 2016) or the manipulation of seed protein composition (Kohno-Murase et al. 1995; Kim et al. 2013; Schmidt et al. 2016) to favour accumulation of protein with higher lysine contents. It should be noted that lysine, aspartate, glutamine and glutamate constitute a central regulatory metabolic network in plant amino acid metabolism (Lam et al. 1995; Zhu and Galili 2003); therefore, directing metabolic flux toward lysine synthesis may impact other pathways. Moreover, glutamate is a major product of the lysine catabolism pathway (Galili et al. 2001) and its homeostasis is strictly regulated because of its vital role in signaling (Forde and Lea 2007). In the current study, the elevated lysine level in the transgenic *C. sativa* seeds expressing *CgDHDPS* was correlated with lower levels of glutamate, although no significant effect on aspartate was observed. A similar observation was made in *A. thaliana* seeds where lysine over-accumulation was positively correlated with glutamine and asparagine, but negatively correlated with aspartate and glutamate (Zhu and Galili 2004). Hence, in any effort to enhance the level of lysine or other essential amino acid it is important to evaluate the overall impact on amino acid composition to ensure that it remains balanced and suitable for the desired application.

Increased accumulation of lysine in *C. sativa* seeds impacted seed production. In plants, lysine synthesis and catabolism are highly regulated, which is in keeping with the important role of lysine in maintaining proper growth and development (Galili 1995; Azevedo and Lea 2001). High levels of free lysine in *N. tobacum* are associated with loss of apical dominance, delayed flowering and senescence, partial sterility and abnormal leaf appearance (Frankard et al. 1992; Shaul and Galili 1993). In *A. thaliana*, constitutively expressing a bacterial *DHDPS* with the *CaMV 35S* promoter in an *lkr/sdh* mutant background caused a dwarf phenotype (Zhu and Galili 2004). Deficiencies of several TCA cycle metabolites, such as fumarate and citrate, leading to energy stress may be responsible for impaired seed germination in this line (Galili 2011; Angelovici et al. 2011). These deleterious effects may be alleviated using a seed-specific promoter to direct expression of the *DHDPS* transgene (Falco et al. 1995). In light of this, the *PAP85* promoter was used in the current study as it is active only during seed maturation and the early phase of seedling growth (Rozwadowski et al. 2008). For the most part, seed germination and early seedling development were not impacted in most of the transgenic lines. However, seed yield was reduced; more so in lines expressing *CgDHDPS* and less so in lines expressing *CsDHDPS mA*. The *CgDHDPS* lines also exhibited higher levels of total seed lysine accumulation and it is possible that this had a deleterious effect on seed development via one or more of the mechanisms described above. The impact on seed yield has not been reported in other similar studies; however, wrinkled seed appearance and poor germination were correlated with higher levels of lysine in soybean lines expressing *CgDHDPS* (Falco et al. 1995). This suggests that there may be practical limits to which lysine levels can be increased in seeds. Based on the range of lysine levels in the transgenic lines and the correlation with the degree of negative impact on growth and development, the practical limit for lysine accumulation in *C. sativa* seed is about a 10% increase above that in the DH55 line (26.74 ± 0.22 mg per g dry defatted meal) used in these experiments. However, it should be noted that we measured total (free and protein-incorporated) lysine as this is an accurate measure of lysine availability. If the negative effects are associated with elevated levels of free lysine, it is possible that inclusion of a more subsuming sink (e.g. a high lysine protein) may alleviate some of these.

In summary, this study demonstrated it is possible to engineer lysine feedback-inhibition insensitive isoforms of *C. sativa* DHDPS resulting in an increase in protein-incorporated lysine in seed. Notably, this study also revealed that individual mutations and combinations of mutations must be examined within the context of the enzyme under study to generate variants that are not only insensitive to lysine, but remain highly active. With the ability to edit genes directly within the *C. sativa* genome (Lyzenga et al. 2019), it may be possible to re-engineer one or more of the endogenous *CsDHDPS* paralogues to confer these properties.

## Supplementary Information

Below is the link to the electronic supplementary material.

Supplementary file1 (TIF 2180 KB)

Supplementary file2 (TIF 2492 KB)

Supplementary file3 (TIF 6344 KB)

Supplementary file4 (DOCX 24 KB)

Supplementary file5 (DOCX 17 KB)

Supplementary file6 (DOCX 12 KB)
