# Supplementary figures and images for: Engineering a feedback inhibition-insensitive plant dihydrodipicolinate synthase to increase lysine content in Camelina sativa seeds
Source: Transgenic Res. 2021 Nov 20;31(1):131–48. doi: 10.1007/s11248-021-00291-6 (PMC8821502; doi:10.1007/s11248-021-00291-6)

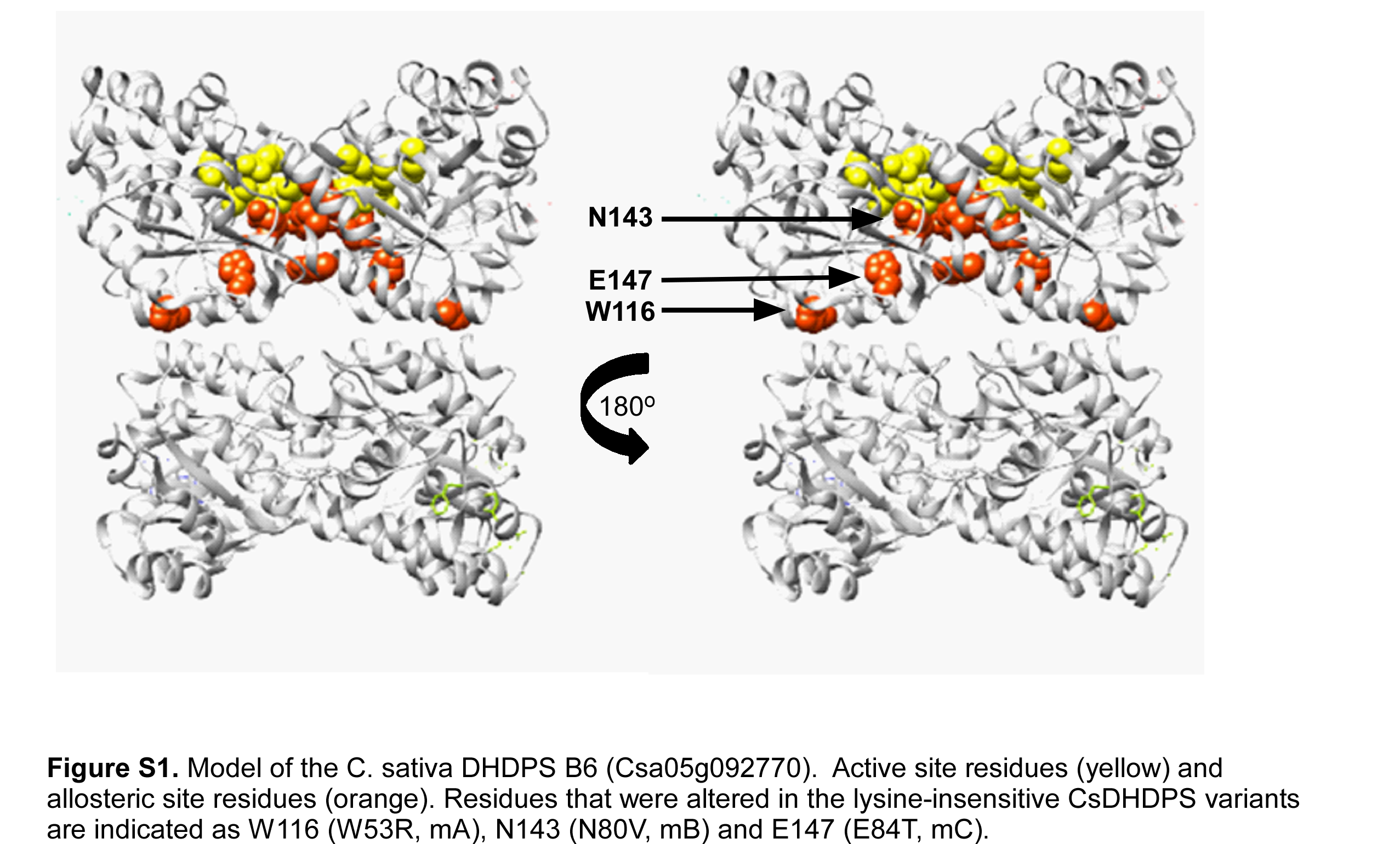

Supplement: Supplementary file 1 — Supplementary file1 (TIF 2180 KB) [file 11248_2021_291_MOESM1_ESM.tif]

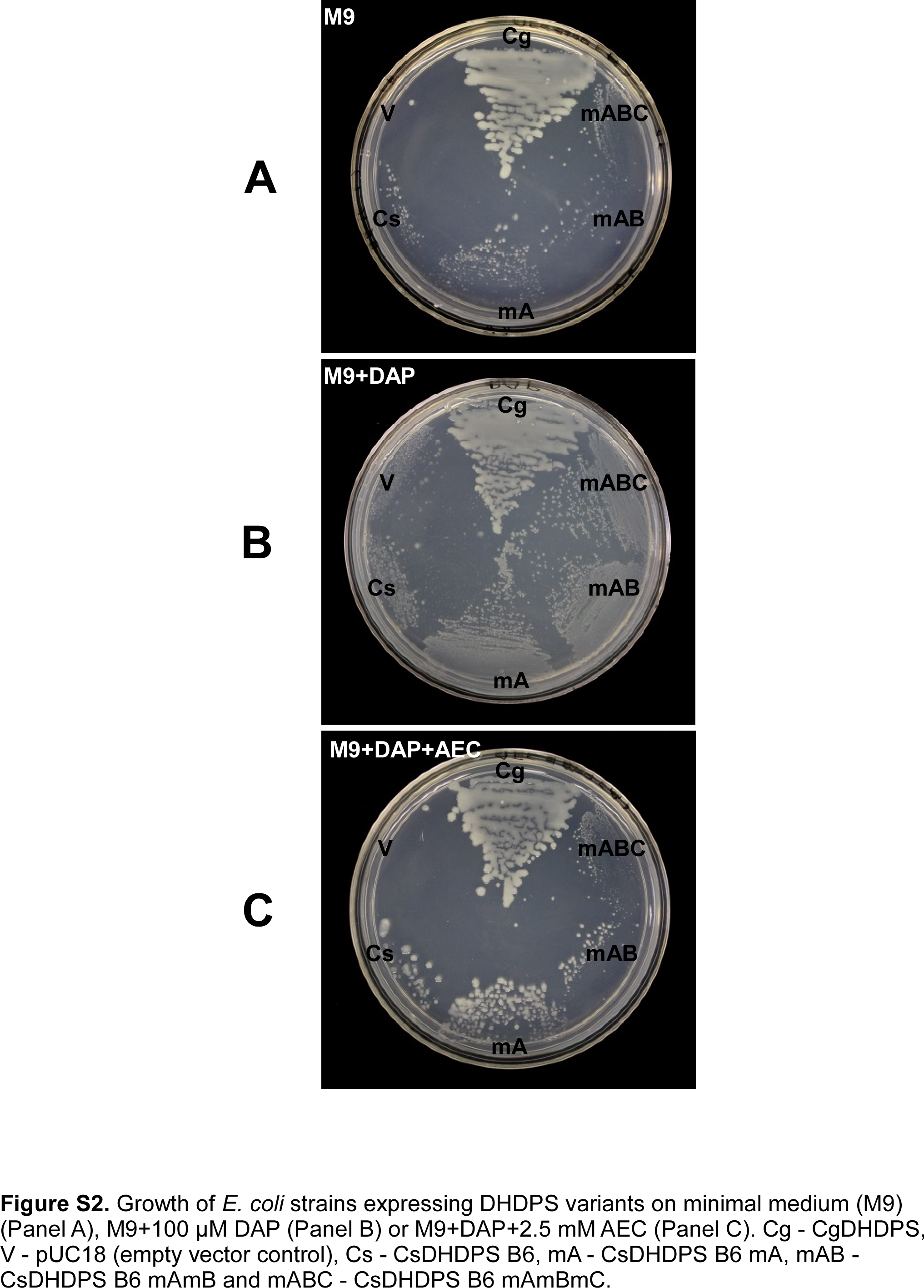

Supplement: Supplementary file 2 — Supplementary file2 (TIF 2492 KB) [file 11248_2021_291_MOESM2_ESM.tif]

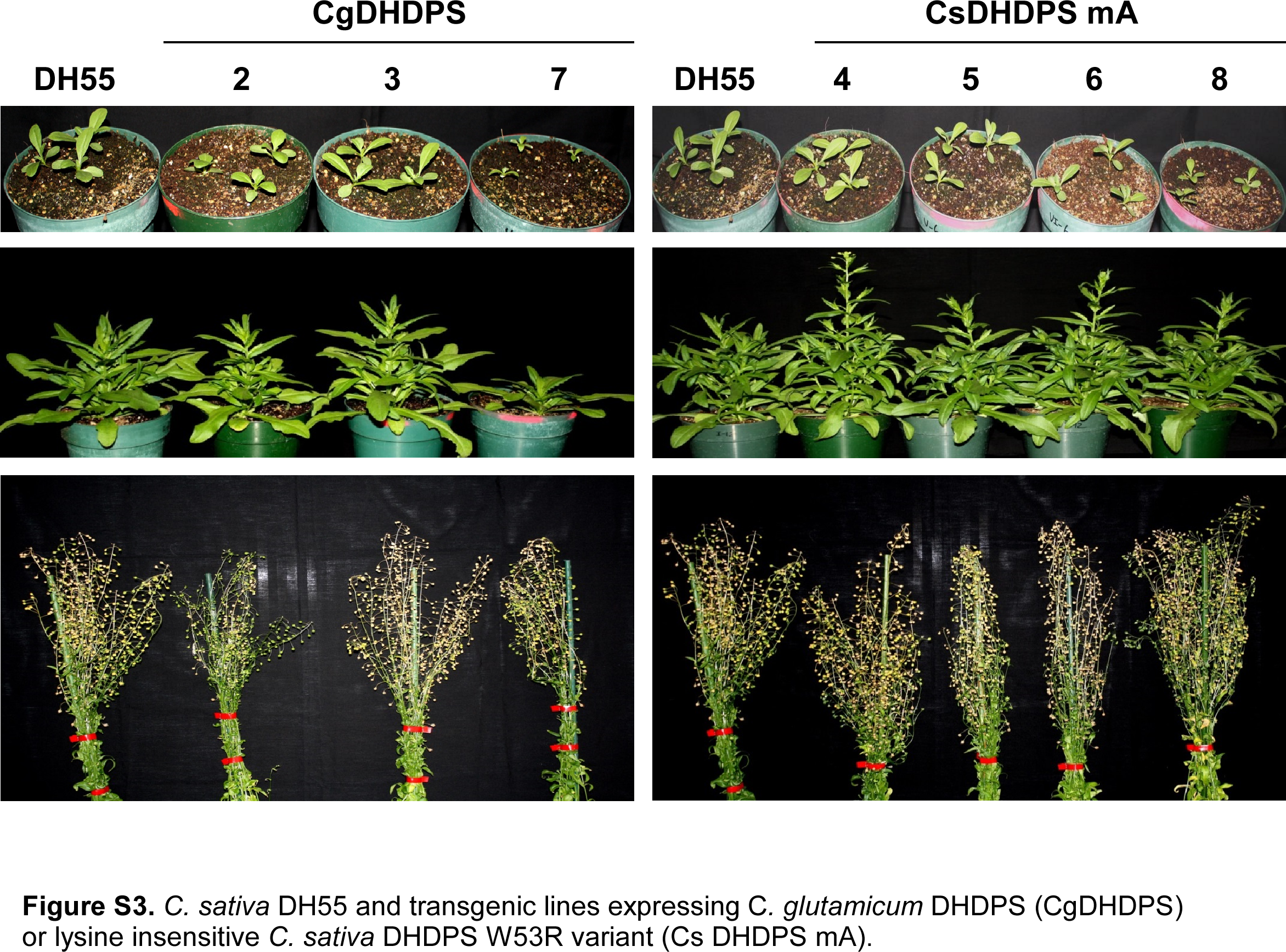

Supplement: Supplementary file 3 — Supplementary file3 (TIF 6344 KB) [file 11248_2021_291_MOESM3_ESM.tif]
