# Supplementary material for: Engineering a feedback inhibition-insensitive plant dihydrodipicolinate synthase to increase lysine content in Camelina sativa seeds
Source: Transgenic Res. 2021 Nov 20;31(1):131–48. doi: 10.1007/s11248-021-00291-6 (PMC8821502; doi:10.1007/s11248-021-00291-6)
Supplement: Supplementary file 4 — Supplementary file4 (DOCX 24 KB) [file 11248_2021_291_MOESM4_ESM.docx]

**Table S1.** Oligonucleotides used in this study. F designates forward primer and R designates reverse primer. Additional sequences are in lower case with restriction enzyme sites in bold. Altered codons are in green.

Name Sequence (5’→3’) Specific Application

--------------------------------------------------------------------------------------------------------------------------------------------------------------------------------------------------

Site Directed Mutagenesis

CSDHDPS-F3 ca**ggatcc**AAGATGGCTGCTTTAAAAGG Amplify and clone *CsDHDPS* ORF into pGEM-T Easy

CSDHDPS-R3 ca**gaattc**CTAATATCGACCGATTAAGATGAAG

CsDHDPS-F6 ca**gaattc**CATGGCAGCTTTGAAAGG Amplify and clone *CsDHDPS* ORF into pUC18

CsDHDPS-R6 ca**ggtacc**CTAATATCGACCGATTAAGATGAAG

W53RB6-F GCCAGTTGATGAGCa**GG**GATGAACACATAATGCTTATAGG SDM of CsDHDPS B6 to introduce TGG→aGG change

W53RB6-R CCTATAAGCATTATGTGTTCATC**CCt**GCTCATCAACTGGC

N80VB6-F GTCATTGGAAACACCGGAAGC**gtC**TCGACTAAAGAAGc SDM of CsDHDPS B6 to introduce AAC→gtC change

N80VB6-R CTTCTTTAGTCGA**Gac**GCTTCCGGTGTTTCCAATGAC

E84TB6-F CCGGAAGCAACTCGACTAAA**acA**GCTATACATGCCACTG SDM of CsDHDPS B6 to introduce GAA→acA change

E84TB6-R CAGTGGCATGTATAGC**Tgt**TTTAGTCGAGTTGCTTCCGG

mBmC-F GGAAACACCGGAAGC**gtC**TCGACTAAA**acA**GCTATACATGCC Create triple mutant W53R/N80V/E84T

mBmC-R GGCATGTATAGC**Tgt**TTTAGTCGA**Gac**GCTTCCGGTGTTTCC

M13-F TTGTAAAACGACGGCCAGT Sequencing insert in pUC18

M13-R GGAAACAGCTATGACCATGA

Binary Vector Construction

CgDHDPS-F ca**gaattc**cATGAGCACAGGTTTAACAGC Amplify *CgDHDPS* locus from *C.* *gluctamicum*

CgDHDPS-R ca**ggtacc**TCATTATAGAACTCCAGC

AtCTP-F ca**gaattc**cATGGCTTCCTCTATGCTC Amplify and clone *A. thaliana* rubisco small subunit CTP

AtCTP-R ca**gaattc**ccGGAATCGGTAAGGTCAGG

CTP-F2 tca**gtcgag**ATGGCTTCCTCTATGCTCTCTTC SOEing PCR to fuse AtCTP into N-terminus of CgDHDPS

CTPCgDS-R1 GTCTTAGCTGTTAAACCTGTGCTCATGGAATCGGTAAGGTCAGGAAGG

CTPCgDS-F1 CCTTCCTGACCTTACCGATTCCATGAGCACAGGTTTAACAGCTAAGAC SOEing PCR to fuse AtCTP into N-terminus of CGDHDPS

CgDS-R2 caGCGGCCGCTTATAGAACTCCAGCTTTTTTCATGTCTTC

CgDHDPS-F1 ca**ggtacc**ATGAGCACAGGTTTAACAGC Cloning and RT-PCR of *CgDHDPS* into pENTR1A

CgDHDPS-R1 ca**gcggccgc**TCATTATAGAACTCCAGC

mCsDHDPS-F11 ca**ggtacc**ATGGCAGCTTTGAAAGG Cloning and RT-PCR of *CsDHDPS-mA* into pENTR1A vector

mCsDHDPS-R11 ca**gcggccgc**CTAATATCGACCGATTAAGATGAAG

PAP85 seq-F GCGTACGAACACTCGTCACC Sequencing insert in recombinant pWY190

Ter seq-R CGGATATAGACATAGCTG

Analysis of Transgenic Plants

PAT-F ACAGACCCACAAACACCAC To access insert copy number

PAT-R GCAATACCAGCCACAACAC

CsACT-F GCTCTTCCATCGAGAAGAACTAC Reference for *C. sativa* actin gene to access copy number

CsACT-R CAAACGAGGGCTGGAATAAGA

Cs18S rDNA-F GGTGGTAACGGGTGACGGAGAA RT-PCR reference to amplify C. sativa 18S cDNA

Cs18S rDNA-R AAGAACGGCCATGCACCACCAC

--------------------------------------------------------------------------------------------------------------------------------------------------------------------------------------------------
