# Supplementary material for: Engineering a feedback inhibition-insensitive plant dihydrodipicolinate synthase to increase lysine content in Camelina sativa seeds
Source: Transgenic Res. 2021 Nov 20;31(1):131–48. doi: 10.1007/s11248-021-00291-6 (PMC8821502; doi:10.1007/s11248-021-00291-6)
Supplement: Supplementary file 5 — Supplementary file5 (DOCX 17 KB) [file 11248_2021_291_MOESM5_ESM.docx]

**Table S2.** Growth Characteristics of *E. coli* strains expressing DHDPS variants

**S2.1.** Time required for bacterial cultures to reach the same density in presence of the lysine analogue AEC.

| *E. coli* Strain | Time (hr) Required to Reach OD 600 = 0.3 per Medium^1^ | | | | | |
| --- | --- | --- | --- | --- | --- | --- |
|  | M9 | M9 + DAP | M9 + AEC  100 | M9 + AEC  300 | M9 + AEC  500 | M9 + AEC  700 |
| AT997 (*dapA*^-^):pUC18 | >108 | 26.3 | >108 | >108 | >108 | >108 |
| AT997 (*dapA*^-^):pUC18+wt*CsDHDPS* | 38.6 | 21.8 | 34.1 | 55.9 | 61.5 | 70.9 |
| AT997 (*dapA^-^*):pUC18+*CsDHDPS-mA* | 29.6 | 24 | 31.8 | 33.3 | 34.3 | 40.4 |
| AT997 (*dapA^-^*):pUC18+*CsDHDPS-mAmB* | 31.3 | 26.4 | 35 | 40 | 41 | 40.5 |
| AT997 (*dapA^-^*):pUC18+*CsDHDPS-mAmBmC* | 31 | 28 | 34 | 36.6 | 36.4 | 39.7 |
| AT997 (*dapA*^-^):pUC18+*CgDHDPS* | 21.8 | 21.2 | 20.6 | 21.1 | 21.1 | 20.7 |

^1^AEC concentration in µm. n=3

**S2.2.** Growth rate of bacterial cultures during logarithmic phase in presence of the lysine analogue AEC.

| *E. coli* Strain | Slope of Logarithmic Phase Line per Medium^1^ | | | | | |
| --- | --- | --- | --- | --- | --- | --- |
|  | M9 | M9 + DAP | M9 + AEC  100 | M9 + AEC  300 | M9 + AEC  500 | M9 + AEC  700 |
| AT997 (*dapA*^-^):pUC18 | 0 | 0.0310 | 0 | 0 | 0 | 0 |
| AT997 (*dapA*^-^):pUC18+wt*CsDHDPS* | 0.0231 | 0.0433 | 0.0216 | 0.0127 | 0.0108 | 0.0076 |
| AT997 (*dapA^-^*):pUC18+*CsDHDPS-mA* | 0.0234 | 0.0377 | 0.0292 | 0.0277 | 0.0320 | 0.0292 |
| AT997 (*dapA^-^*):pUC18+*CsDHDPS-mAmB* | 0.0352 | 0.0363 | 0.0282 | 0.0372 | 0.0375 | 0.0417 |
| AT997 (*dapA^-^*):pUC18+*CsDHDPS-mAmBmC* | 0.0392 | 0.0348 | 0.0336 | 0.0280 | 0.0336 | 0.0350 |
| AT997 (*dapA*^-^):pUC18+*CgDHDPS* | 0.0465 | 0.0425 | 0.0468 | 0.0467 | 0.0439 | 0.0427 |

^1^AEC concentration in µm. n=3
