# Supplementary material for: Engineering a feedback inhibition-insensitive plant dihydrodipicolinate synthase to increase lysine content in Camelina sativa seeds
Source: Transgenic Res. 2021 Nov 20;31(1):131–48. doi: 10.1007/s11248-021-00291-6 (PMC8821502; doi:10.1007/s11248-021-00291-6)
Supplement: Supplementary file 6 — Supplementary file6 (DOCX 12 KB) [file 11248_2021_291_MOESM6_ESM.docx]

**Supporting information**

**Table S1.** Oligonucleotides used in this study.

**Table S2.** Growth Characteristics of *E. coli* strains expressing DHDPS variants.

**Table S3.** Amino acid composition in dry seed of DH55 and transgenic lines expressing either *CgDHDPS* or *CsDHDPS-mA* cDNA (mean±S.E., n=3). Different letters following the values denote significant difference (*p*<0.05) between DH55 and the transgenic line.

**Figure S1.** Model of the *C. sativa* DHDPS B6 (Csa05g092770). Active site residues (yellow) and allosteric site residues (orange). Residues that were altered in the lysine-insensitive CsDHDPS variants are indicated as W116 (W53R, mA), N143 (N80V, mB) and E147 (E84T, mC).

**Figure S2.** Growth of *E. coli* strains expressing DHDPS variants on minimal medium (M9), M9+100 µM DAP or M9+DAP+2.5 mM AEC. Cg - CgDHDPS, V- pUC18 (empty vector control), Cs - CsDHDPS B6, mA - CsDHDPS B6 mA, mAB - CsDHDPS B6 mAmB and

mABC - CsDHDPS B6 mAmBmC. Plates were incubated at 37 ^o^C for approximately 144 hr.

**Figure S3.** *C. sativa* DH55 and transgenic lines expressing *C. glutamicum* DHDPS (CgDHDPS) or lysine insensitive *C. sativa* DHDPS W53R variant (Cs DHDPS mA).
